# BCL-xL is correlated with disease severity in neonatal infants with early sepsis

**DOI:** 10.1186/s12887-021-02764-3

**Published:** 2021-06-30

**Authors:** Wu Wenshen, Peng Qi, Huang Tianli, Liao Jinfeng, Li Ning

**Affiliations:** 1 Neonatal Department, Dongguan Children’s Hospital, Guangdong Medical University, NO.68, Xihu the Third Road, Shilong Town, Guangdong Pronvince Dongguan City, China; 2 Dongguan Institute of Pediatrics, NO.68, Xihu the Third Road, Shilong Town, Guangdong Pronvince Dongguan City, China

**Keywords:** Neonatal critical illness score, BCL-xL, Sepsis, Receiver operator characteristic curve

## Abstract

**Background:**

Sepsis is the most common cause of morbidity and mortality in neonatal infants. It is essential to find an accurate and sensitive biomarker to confirm and treat neonatal sepsis in order to decrease the rate of mortality. The aim of this study was to investigate the association between disease severity in patients with sepsis and TNF-α, B cell lymphoma-extra-large (BCL-xL), and serum Mitochondrial membrane potential (MMP).

**Methods:**

We investigated the correlation between SNAP-II score and levels of TNF-α, BCL-xL, and MMP-index, respectively. The receiver-operating characteristics (ROC) was to assess the diagnostic value of the the Bcl-xL in the diagnosis of the of septic shock.

**Results:**

A total of 37 infants were diagnosed with sepsis. SNAP-II was positively correlated with the level of BCL-xL (*r* = 0.450, *P* = 0.006). The area under the BCL-xL curve was 83.0 %, and the 95 % CI was 67.1–93.3 %. The septic shock threshold was > 3.022 ng/mL, and the sensitivity and specificity were 75.0 and 95.2 %, respectively. The positive predictive value was 92.3 %, and the negative predictive value was 83.3 %. Furthermore, the level of SNAP-II was > 10, and BCL-xL was > 3.022 ng/mL as the threshold, and the sensitivity, specificity, positive predictive value, and negative predictive value of septic shock were 93.8 %, 95.2 %, 93.8 %, and 95.2 %, respectively.

**Conclusions:**

BCL-xL is associated with the progression of sepsis. The combination of BCL-xL and SNAP-II could be early predicte the severity of the disease.

## Introduction

Sepsis is the most common cause of morbidity and mortality in neonatal infants, thus rendering a large global sepsis burden in neonates [1]. The reported overall neonatal sepsis rate ranges from 1 to 5 cases per 1000 live births [1, 2]. The clinical manifestations of sepsis in neonates are nonspecific and varied [3]. Occasionally, the clinical signs and symptoms have a delayed onset, leading to refractory sepsis in the neonates. The gold standard for the diagnosis of sepsis is blood culture; however, it has low sensitivity in neonates because of maternal antibiotic therapy, low or intermittent bacteremia, and small volumes of blood samples [4]. Therefore, finding accurate and sensitive biomarkers to confirm and treat neonatal sepsis is essential to decrease the rate of mortality. Tumor necrosis factor-alpha (TNF-α) is a proinflammatory cytokine that is produced during systemic infection and inflammation. The level of TNF-α is higher in septic newborns as compared to healthy newborns [3]. Mitochondrial membrane potential (MMP) in platelets is correlated with the disease severity in sepsis patients [5]. Moreover, platelets play a key role in fighting infections. Conversely, bacteria can directly activate the apoptotic pathway in platelets to induce platelet cell death in vitro [6]. Apoptosis is a tightly regulated biological process that plays a central role in sepsis and in the pathophysiology of septic complications [6]. Thus, the present study aimed to investigate the association between disease severity in patients with sepsis and TNF-α, B cell lymphoma-extra-large (BCL-xL), and serum MMP.

## Methods

### Study population

The study was conducted from January 2016 to December 2017 in a Level III NICU, which has 30 beds and admits approximately 800 patients per year. The study was approved by the Dongguan Children’s Hospital, and written consent was obtained from neonates’ parents. The neonates with evidence of sepsis were enrolled in this study.

Patients with septic shock within 24 h were enrolled in the septic shock group, while those without shock were enrolled in the sepsis group. Follow-up was performed to observe 28-day mortality. Septic shock was diagnosed if an infant suffered from shock in addition to evidence of sepsis [3, 4]. Shock is defined as the presence of either or both of the following criteria: (1) Systolic (SBP) or diastolic blood pressure (DBP) < 5th percentile for the post-menstrual age. (2) Presence of two or more of the following: Capillary refill time > 3 s, feeble pulse, core to periphery temperature difference > 3 °C, urine output < 0.5 mL/kg/h, base excess >–5.0 mmol/L, or serum lactate > 5 mmol/L. Sepsis was diagnosed if either or both of the following criteria were met [2]: (1) Blood or/and cerebrospinal fluid culture was positive. (2) Any two of the following sepsis screen variables were positive: C-reactive protein > 10 mg/L, microerythrocyte sedimentation rate > 10 mm after the first hour, total leukocyte, and absolute neutrophil counts were beyond the reference range, or immature to total neutrophil ratio was > 0.2. Neonates with complex congenital heart disease and congenital organ malformation were excluded from the study.

### SNAP-II

The Score for Neonatal Acute Physiology-II (SNAP-II) was used to evaluate the severity of the disease. SNAP-II was calculated based on six variables for each infant during the first 12 h of admission [2] (Table 1).

**Table 1:** Each variable point for SNAP-II

Variable	SNAP-II
Mean blood pressure	
>29mmHg	0
20-29 mmHg	9
<20 mmHg	19
Lowest temperature	
>36.5 °C	0
35-36.5 °C	8
>36.5°C	15
PO^2^/FiO_2_ ratio	
>2.49	0
1.0-2.49	5
0.33-0.99	16
<0.33	28
Lowest blood pH	
>7.19	0
7.10-7.19	7
<7.10	16
Multiple seizures	
No	0
Yes	19
Urine output	
>0.9 ml/kg/h	0
0.1-0.9 ml/kg/h	5
<0.1 ml/kg/h	18
Birth weight	
>999 g	-
750-999 g	-
<750 g	-
Small for gestational age	
>3^rd^ percentile	-
<3^rd^ percentile	-
APGAR score at 5min	
>=7	-
< 7	-
Maximum score	115

*SNAP-II* the Score for Neonatal Acute Physiology-II

### Blood samples preparation

Blood samples were obtained at the time point of admission to NICU. 4 ml of blood were sampled from infants to measure MMP. 2 ml of blood were sampled from infants to measure TNF-α and BCL-xL. Serum MMP samples were collected into EDTA-treated tubes and assessed by flow cytometry. Serum samples for measuring the concentrations of TNF-α and BCL-xL were collected by centrifugation of the blood samples at 1,000 ×*g* for 15 min after allowing to clot for 30 min and stored at -40 °C for subsequent assays.

### MMP by flow cytometry

Serum MMP was measured using JC-1 dye (Invitrogen, Carlsbad, CA) and flow cytometry (Immunochemistry Technologies, Bloomington, MN, USA). Samples were diluted to 3 × 10^7^ platelets/mL and stained with JC-1 for 20 min in the dark. MMP was assessed as “MMP-index,” which is a ratio of the mean FL2 (red fluorescence) and FL1 (green fluorescence) [7]. The change in the MMP-index reflects the mitochondrial functional states. Therefore, a decrease in the MMP-index represents a loss in MMP [7].

### Serum TNF-α and BCL-xL analysis

The serum TNF-α and BCL-xL concentrations were measured using a commercial enzyme-linked immunosorbent assay (ELISA) kit (Cusabio Biotech Co., Ltd, Wuhan, China), according to the manufacturer’s instructions. Detection limit for serum TNF-α level was 1.95 pg/mL, measure range was 7.8–500 pg/mL. Detection limit for serum BCL-xL level was 0.039 ng/mL, measure range was 0.156–10 ng/mL. Both the TNF-α and BCL-xL absorbance values of the standards and samples were obtained at 450 (reference wavelength 540–570) nm using a Multiskan MK3 spectrophotometer (Thermo Scientific, Waltham, MA, USA).

### Statistical analysis

All statistical analyses were performed using SPSS version 23 (IBM Co., Armonk, NY, USA). Continuous variables were expressed as median and interquartile ranges. The categorical variables were summarized as counts and percentages. Spearman’s correlation analysis was used to investigate the correlation between SNAP-II and levels of TNF-α, BCL-xL, and MMP-index, respectively. The receiver operating characteristic (ROC) curve was used to calculate the diagnostic value, the area under the curve (AUC) and 95 % confidence interval (CI) were analyzed. The sensitivity, specificity, positive predictive value, and negative predictive value were calculated. *P* < 0.05 was considered statistically significant.

## Results

A total of 37 infants were diagnosed with sepsis. The age of admission to NICU was 1 (range, 0.5–456) hour. The gestational age of the cohort was 39 (range, 37–40) weeks, and the birth weight was 3.00 (range, 2.70–3.32) kg. Sixteen (43.2 %) infants developed septic shoc’k within 24 h in the hospital. After 28 days of follow-up, 3 patients died and the mortality rate was 18.8 % in the septic shock group and none patient died in the sepsis group. Of the 37 neonates, 7(18.9 %)had a positive blood culture result. Among cultures with gram-positive species, *Staphylococcus hominis* (*n* = 2), *Raoultella terrigena* (*n* = 1), *enterobacter cloacae (n = 1), Escherichia coli* (*n* = 1), *Nearly smooth Candida*, (*n* = 1) and *Staphylococcus epidermidis* (*n* = 1) were found. Seven (43.8 %) infants had a positive blood culture result in the septic shock and none in the sepsis group. There were not any difference of the demographics characteristics between culture positive and culture negative infants. After 28 days of follow-up, three infants died in the septic shock group and none patient died in the sepsis group.

The demographics and diagnostic characteristics are summarized in Table 2.

**Table 2:** The general characteristics of patients

Variable	N (total = 37)
Birth weight	
Median (IQR) - g	3000 (2665, 3345)
Distribution – n(%)	
<1500 g	0 (0%)
1500–2500 g	8 (21.6%)
>2500 g	29 (78.4%)
Sex - n(%)	
Male	25 (67.6%)
Female	12 (32.4%)
Gestational age	
Median (IQR) - wk	39 (37, 40)
Distribution – n(%)	
<37 wk	7 (18.9%)
>=37 wk	30 (81.1%)
Mode of delivery - n(%)	
Vaginal	15 (40.5%)
Cesarean section	22 (59.5)
Postnatal age (IQR) - h	2 (1, 3)
Sepsis type - n (%)	
Culture proven	7 (18.9%)
Screen positive	30 (81.1%)
Respiratory support - n (%)	
None	0 (0%)
nCPAP	17 (45.9%)
MV	20 (54.1%
SNAP-II (IQR)	10 (10, 23)
Duration of hospital stay ( IQR) - days	
Survival	5 (4, 16)
non-Survival	2 (4, 16)
Time death ( IQR) - days	
Outcomes - n (%)	
Died	3 (8.1%)
Survived	34 (91.9%)

*MV* mechanical ventilation, *nCPAP* nasal continuous positive airway pressure, *SNAP-II* the Score for Neonatal Acute Physiology-II

The SNAP-II level was measured in 37 (100 %) patients with a median of 10 (10–23). BCL-xL, TNF-α, and MMP-index were detected in 36 patients (97.3 %), 34 patients (91.9 %), and 11 patients (29.7 %), respectively. The median of BCL-xL, TNF-α, and MMP-index was 1.39 (0.76–5.69) ng/mL, 88.90 (64.22–110.46) pg/mL, and 0.43 (0.21–0.64), respectively.

**Fig. 1:**
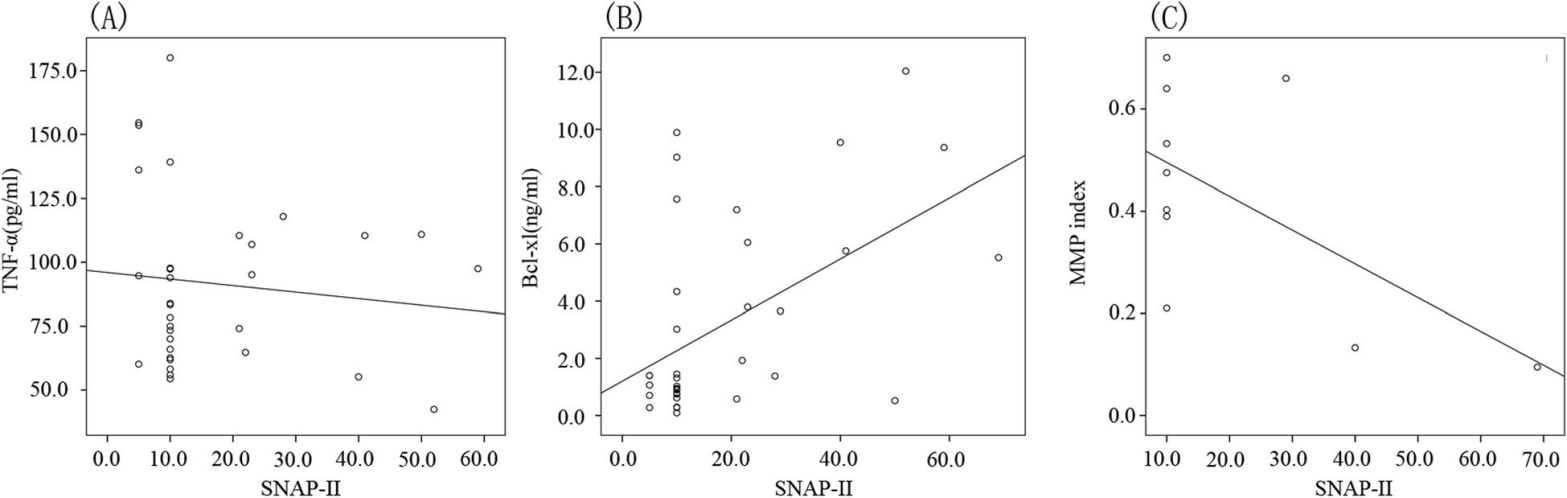
**A** SNAP-II was poorly negatively correlated with TNF-α (*r* = − 0.073, *P* = 0.681) ; **B **SNAP-II was positively correlated with the level of BCL-xL (*r* = 0.450, *P* = 0.006); **C** SNAP-II was poorly negatively correlated with MMP-index (*r* = − 0.455, *P* = 0.187). SNAP-II: the Score for Neonatal Acute Physiology-II, MMP: Mitochondrial membrane potential

We used Spearman’s correlation analysis to investigate the correlation between clinical disease score (SNAP-II) and levels of BCL-xL, TNF-α, and MMP-index in patients with sepsis. SNAP-II was poorly negatively correlated with TNF-α (*r* = − 0.073, *P* = 0.681; Fig. 1A) and MMP-index (*r* = − 0.455, *P* = 0.187; Fig. 1C) but positively correlated with the level of BCL-xL (*r* = 0.450, *P* = 0.006; Fig. 1B).

**Fig. 2:**
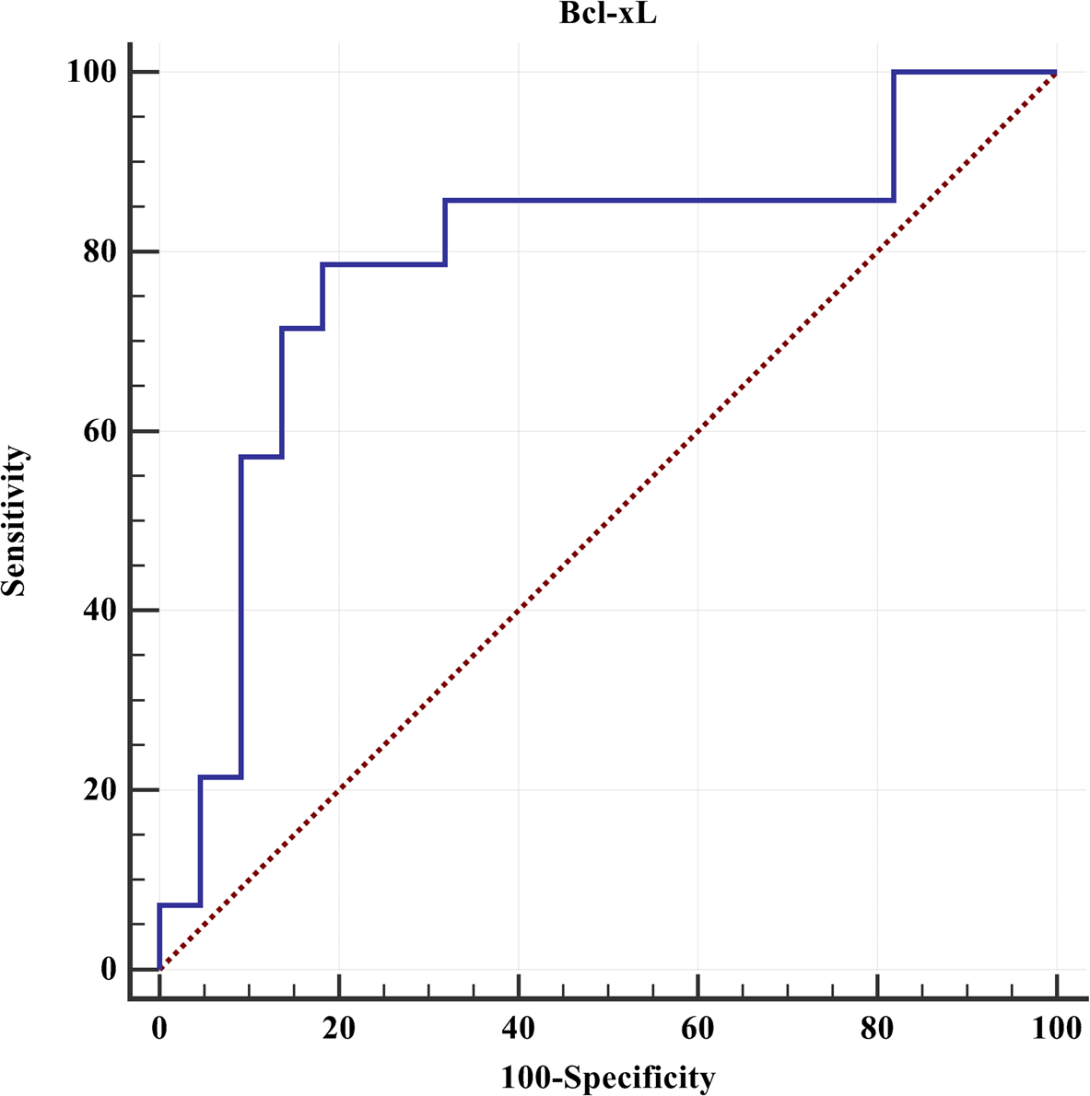
The receiver operating characteristic curve of septic shock and Bcl-xL

BCL-xL was used to analyze the severity of sepsis. The median level of BCL-xL in the septic shock group was 5.64 (range, 1.95–8.66) ng/mL, which was higher than that in the septic group 0.93 (0.66–1.40) ng/mL (*P* < 0.001). The area under the BCL-xL curve was 83.0 %, and the 95 % CI was 67.1–93.3 % (Fig. 2). The septic shock threshold was > 3.022 ng/mL, and the sensitivity and specificity were 75.0 and 95.2 %, respectively. The positive predictive value was 92.3 %, and the negative predictive value was 83.3 %. Furthermore, the level of SNAP-II was > 10, and BCL-xL was > 3.022 ng/mL as the threshold, and the sensitivity, specificity, positive predictive value, and negative predictive value of septic shock were 93.8 %, 95.2 %, 93.8 %, and 95.2 %, respectively.

In 10 patients (27.0 %) who had MMP, it was noted that the lower the MMP, the worse outcomes in neonatal sepsis. A patient with a prolonged hospital stay (up to 90 days) had the lowest MMP (0.095), and a patient was eventually deceased had the second-lowest MMP (0.133).

## Discussion

Sepsis is a leading cause of morbidity and mortality for children worldwide [1]. The majority of children who die of sepsis suffer from refractory shock and/or multiple organ dysfunction syndrome (MODS) within the initial 72 h of the treatment [8, 9]. In addition, sepsis has a prolonged hospitalization duration and high treatment costs, are major concerns to human health [8, 9]. Therefore, early identification and appropriate resuscitation and management are critical to optimizing the outcomes for infants with sepsis. In this study, we demonstrated that the levels of BCL-xL, but MMP-index and TNF-α, were significantly correlated with disease severity (SNAP II).

The BCL-xL belongs to the Bcl-2 family and regulates cell apoptosis. In the presence of an apoptosis signal, BCL-xL is translocated to the mitochondrial outer membrane. Also, it is distributed on the cytoplasm and membrane in normal conditions [10]. BCL-xL inhibits not only cell apoptosis [11] but also cell necrosis [12]. It is a protective protein against cell damage induced by an inflammatory response in the body.

In a study of adult sepsis [5], the content of BCL-xL and platelet MMP in patients with septic shock was decreased at the same time as compared to those with sepsis, which indicated that the mitochondrial function was severely damaged, and the normal oxidative phosphorylation of the cell could not be carried out. This resulted in a critical state of the body, which was in agreement with the critical disease score. Currently, there are no data on the BCL-xL serum content. We speculated that the expression of BCL-xL protein in the cells was enhanced under the stimulation of inflammatory factors before sepsis. However, after the cells and mitochondria were severely damaged and could not maintain normal morphology and function, the generated BCL-xL protein was released into the blood. Therefore, we aimed to provide effective and reliable molecular biological indicators for clinicians to assess the degree of damage and the severity of the disease and predict the disease progression through the detection of BCL-xL level in the blood of newborns with sepsis. In this study, the level of BCL-xL was significantly correlated with disease severity, reflecting the interaction between external damage and the body, as well as the severity of the disease. Both the sensitivity and specificity of the BCL-xL were in distinguishing sepsis shock. The blood BCL-xL content is expected to be a new molecular marker to identify sepsis shock early.

A correlation has been established between SNAP II and the severity outcome. In this study, SNAP II in the septic shock group was significantly higher than that in the sepsis group. Blood BCL-xL protein level was similar to that of SNAP-lI. These findings suggested that the combination of BCL-xL and SNAP-II was more sensitive and specific than the BCL-xL to predict neonatal sepsis outcomes.

Sepsis is considered to be over-inflammation, followed by protracted inflammation and immune suppression [13]. TNF-α regulates apoptosis by caspase-8 and Fas apoptotic pathway [14]. Mitochondria constitute the central target in the apoptotic pathway leading to platelet apoptosis, and mitochondrial damage is an early indicator of cell death in platelets. A previous study demonstrated a correlation between platelet MMP-index and disease severity in ICU patients with Systemic Inflammatory Response Syndrome (SIRS) [6]. In this study, patients did not show a significant correlation between MMP-index and TNF-α with SNAP-II. SNAP-II has high sensitivity and specificity in predicting severity outcomes in neonatal sepsis [15, 16], but another study demonstrated that the value of SNAP-II was low for predicting mortality [5]. In the current study, most cases were mild: 24 (64.9 %) patients had SNAP-II < 20. Simonson et al. [16] reported that at a cut-off value of ≥ 20 in the presence of sepsis, the SNAP-II score could predict the mortality outcome.

Nevertheless, the present study has several limitations. First, the sample size of 37 patients is small. Thus, large clinical trials are needed to confirm the current findings. Secondly, while most patients (73.0 %) did not obtain an MMP-index, only 10 patients (27.0 %) showed the index. This is due to the high requirement of the MMP-index examination of fresh blood specimens at the earliest. Many patients failed to achieve MMP because they missed the optimal examination time, which leads to the failure of experiments. The next step is to improve the detection rate of MMP-index.

We demonstrated that BCL-xL was enhanced in circulating platelets in patients with sepsis. Thus, the findings suggested that BCL-xL is associated with the progression of sepsis. The serum BCL-xL combined with SNAP-II could be early predicte the severity of the disease.

## Data Availability

The datasets used and analysed during the current study available from the corresponding author on reasonable request.
